# Towards leprosy elimination by 2020: forecasts of epidemiological indicators of leprosy in Corrientes, a province of northeastern Argentina that is a pioneer in leprosy elimination

**DOI:** 10.1590/0074-02760160490

**Published:** 2017-06

**Authors:** Elisa Petri de Odriozola, Ana María Quintana, Victor González, Roque Antonio Pasetto, María Eugenia Utgés, Octavio Augusto Bruzzone, María Rosa Arnaiz

**Affiliations:** 1 Programa Provincial para el Control de Leishmaniasis y Lepra, Corrientes, Argentina; 2 Programa Nacional para el Control de Tuberculosis y Lepra, Argentina; 3 Centro de Diagnóstico e Investigación en Endemo-Epidemias, Ciudad Autónoma de Buenos Aires, Argentina; 4 Instituto Nacional de Tecnología Agropecuaria, Río Negro, Argentina

**Keywords:** leprosy, epidemiology, infectious disease, forecast, Mycobacterium leprae

## Abstract

**BACKGROUND:**

Corrientes, a province of northeastern Argentina with endemic leprosy, has improved its epidemiological indicators, however, a study of the dynamics over time is lacking.

**OBJECTIVES:**

We analysed data of 1308 leprosy patients between 1991 to 2014, and the forecast for 2020.

**METHODS:**

Descriptive statistics and stepwise Bayesian model selection were performed. Forecasts were made using the median of 100,000 projections using the parameters calculated via Monte Carlo methods.

**RESULTS:**

We found a decreasing number of new leprosy cases (-2.04 cases/year); this decrease is expected to continue by an estimated 20.28 +/- 10.00 cases by 2020, evidenced by a sustained decline in detection rate (from 11 to 2.9/100,000 inhabitants). Age groups that were most affected were 15-44 (40.13%) and 45-64 (38.83%) year olds. Multibacillary forms (MB) predominated (70.35%) and while gradually declining, between 10 and 30% developed disability grade 2 (DG2) (0.175 (0.110 - 0.337) DG2/MB cases), with a time delay between 0 to 15 years (median = 0). The proportion of MB clinic forms and DG2 increased and will continuously increase in the short term (0.036 +/- 0.018 logit (MB/total of cases).

**MAIN CONCLUSIONS:**

Corrientes is on the way to eliminating leprosy by 2020, however the increased proportion of MB clinical forms and DG2 signals a warning for disease control efforts.

Leprosy is a chronic infectious disease caused by *Mycobacterium leprae* that has affected humanity since ancient times, with citations already seen in books of the Vedas and from China between the years 500 and 300 BC. The disease came to America with the Spanish conquest and in the Viceroyalty of the Río de la Plata, present-day Argentina, the first four cases were described in 1792 (de las Aguas 2006). Globally, this disease is considered to persist as a result of prevailing social inequality, affecting poor and marginalised populations and causing social stigma and discrimination (van Brakel et al. 2012). However, there appears to be insufficient evidence to affirm this as fact (Schreuder et al. 2016).

Worldwide, leprosy has mainly been reported in countries in intertropical zones, with 80% of all new cases of leprosy held in India, Brazil, Indonesia, Bangladesh, and Ethiopia (Schreuder et al. 2016, WHO 2016). In the Regions of Latin America and the Caribbean, of the 24 countries that reported new cases of leprosy in 2006, all except Brazil, have reached the elimination target proposed by the World Health Organization (< 1 case/10,000 inhabitants) at the national level (WHO 1991). Argentina, a country that reports around 400 new cases of leprosy per year, reached this goal in 2011 when the region was studied as a whole, but the disease distribution is not uniform throughout the country (WHO 2016). The climate is characterised by great variability due to the extensive land surface and the longitudinal and latitudinal range, which includes several geoastronomical zone (http://www.smn.gov.ar). Since 1996, a high burden of leprosy has been reported in the northeastern region of Argentina, including the provinces of Entre Ríos, Misiones and Corrientes, in addition, Santiago del Estero, Buenos Aires and its suburbs (Escalada & Bonano 1996). In the southern region of the country, which is characterised by low temperatures, the burden of leprosy is very low and new reported cases are due to temporary labour migration. In this context, the level of endemicity of leprosy in Argentina is based on a comparison between provinces with the highest and the lowest number of new cases of the disease.

Corrientes is a province that is surrounded by the Paraná and Uruguay rivers and has a subtropical warm climate with no dry season, with an annual rainfall that ranges from 1,100 and 1,900 mm and sharing part of a humid subtropical forest biome with southern Brazil and Paraguay (Cabrera 1976). Although Corrientes was one of the first provinces to pass a Law of Mandatory Reporting of Leprosy cases (SAIJ 1926) in 1926 and that national Law (LSN 1983) guarantees free and compulsory treatment of leprosy in all official establishments since 1983, the disease remains a public health problem. However, there are no epidemiological or statistical records to support this claim. Within the framework of the policies on health protection and access to health services, the Ministry of Health of the Nation unified the control plan for tuberculosis and leprosy, creating in 2014 the National Program for Control of Tuberculosis and Leprosy (PNCTByL 2014) (Res. 583/14). The PNCTByL and the Provincial Programs for the Control of Leishmaniasis and Leprosy, hold an annual joint meeting where the national situation regarding leprosy is outlined.

This study aimed to investigate the dynamics of the epidemiological indicators of leprosy in the province of Corrientes utilising a 24-year dataset, and what it is forecast to be by 2020.

## MATERIALS AND METHODS

**Fig. 1:**
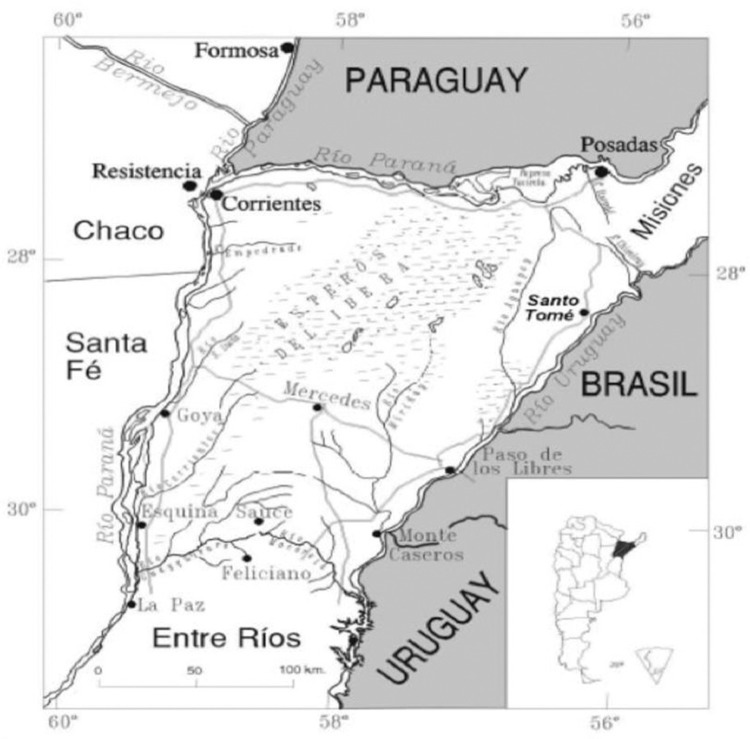
: map of Argentina and the province of Corrientes. Corrientes borders with the Republic of Paraguay, Brazil and Uruguay.

*Area and study population* - The province of Corrientes is located between 27º15’ and 30º44’S, and 55º40’ and 59º 37’W. It has a population density of 11.3 inhabitants/km^2^ living in an area of 88.886 km^2^ and is organised into 25 departments and 70 municipalities (the minimum administrative unit) (deyc-corrientes.gov.ar/) (Fig. 1).

With a population of 992,595 million (deyc-corrientes.gov.ar/), the health system of Corrientes is organised into 64 CAPS (primary level of attention), grouped in five sanitary zones (Ministerio de Salud Pública, Corrientes), with a regional reference clinic for leprosy care, the Dermatological Clinic Hersilia C de Blaquier (secondary level of attention).

*Methodological design* - This was a retrospective and quantitative epidemiological study of 1308 leprosy patients. Data were obtained from the medical records at the Dermatological Clinic Hersilia C de Blaquier of Corrientes. The Dermatological Clinic was founded in 1932 for the care leprosy patients and since 1984 it has been the reference centre for clinical diagnosis, laboratory testing and treatment of leishmaniasis and leprosy, as well as the head of the Provincial Program for the Control of Leishmaniasis and Leprosy for Corrientes. Professionals from this clinic provide care to 16,000 patients each year in addition to carrying out health education and training activities throughout the province. The dermatological clinic gathers information on all new cases of leprosy diagnosed in the province, either through passive detection (voluntary communication and notification) or active detection (household contacts and queries). The future course of the leprosy variables was analysed in order to predict the evolution of this endemic disease out to the year 2020.

*Diagnosis* - The diagnosis of leprosy was based on clinical examination, histopathological findings and tissue smears. Biopsies were analysed in the Pathology Department of the Teaching Hospital Gral José de San Martín (City of Corrientes) and Ziehl-Neelsen staining was performed on tissue smears from the ear lobes, nasal mucus and skin to identify acid-fast bacilli consistent with *M. leprae*. Patients were classified into two major groups: paucibacillary (PB), associated with less than five skin lesions and/or smear negative (-) and multibacillary (MB), associated with more than five skin lesions and/or smear positive (+) (WHO 1982).

*Study variables and analyses methods: raw data extracted from medical records* - The raw data extracted from the medical records included: (i) new cases of leprosy diagnosed from 1991 to 2014; (ii) new cases of leprosy classified by age group (0-14 years, 15-44 years, 45-64 years and over 65 years); and (iii) new cases of leprosy according the clinical forms (PB or MB), which takes into account the therapeutic scheme used (WHO 1982).

*Variables calculated from raw data* - (i) Proportion of MB cases of leprosy among all new cases detected during the year, in a time series between 1991 and 2014 (WHO 2009); (ii) proportion of new cases of leprosy with Grade 2 disabilities (DG2) among all new cases detected during the year, in a time series between 1991 and 2014 (WHO 2009).

*Statistical analyses: descriptive statistics* - The annual detection rate of new cases of leprosy was calculated by dividing the number of new cases diagnosed per year by the total population of residents of Corrientes, multiplied by 100,000 [DEyC de Corrientes, 1991, 2001, 2010 (Available from: deyc-corrientes.gov.ar/)]. The annual detection rate of new cases of leprosy by age group (0-14 years, 15-44 years, 45-64 years and over 65 years) was calculated by dividing the number of new cases of each age group per year by the healthy population of each group in the same period estimated by the census of 1991, 2001 and 2010, multiplied by 100,000 (deyc-corrientes.gov.ar/).

The prevalence rate was calculated by dividing the number of new cases of leprosy per year undergoing treatment by the total resident population of Corrientes in the same period, estimated by the census of 1991, 2001 and 2010, multiplied by 10,000 (deyc-corrientes.gov.ar/).

*Data analysis* - To understand the epidemiological dynamics in the studied province, the data series obtained was analysed by means of a stepwise Bayesian model selection procedure (Gelman et al. 2004). In this procedure, we started from the simplest possible model (a null or constant model), with only one or two parameters, and added new parameters according to the biological hypothesis being tested, until the increase in the complexity of the model (measured by the number of parameters) exceeded the improvement in its explanatory power (in terms of log-likelihood function). The deviance information criterion (DIC) was choosen, according to Gelman et al. (2004), as a measure of the explanatory power-complexity compromise (Table).

**TABLE:** Deviance information criterion (DIC) values of the tested models

a: Total cases proposed models		
Model	#Parameters	DIC
Null model	2	233.22
Autoregressive (AR)	2	274.90
Moving average (MA)	2	299.76
Linear trend	3	194.86*
Quadratic trend	4	196.22
AR + MA	3	275.78
LTM + AR	3	195.67
LTM + MA	3	196.01
LTM + AR + MA	4	196.33
b: Multibacillary proposed models		
Null model	1	140.52
Autoregressive (AR)	2	136.91
Moving average (MA)	2	138.13
Logit trend model (LTM)	2	127.52*
AR + MA	3	136.18
LTM + AR	3	129.62
LTM + MA	3	128.42
LTM + AR + MA	4	129.09
c: DG2 proposed models		
Null model	2	133.03
Lineal multibacillary (LMB)	3	124.45
Lineal time	3	123.87
Autoregressive (AR)	3	139.57
Moving average (MA)	3	139.59
LMB + Time	4	123.49
Time + AR	4	138.91
Time + MA	4	143.61
LMB + AR	4	126.68
LMB + MA	4	126.30
LMB + Delay	4	125.20
LMB + Time + Delay	5	124.70
LMB + Time + AR	5	125.60
LMB + Time + MA	5	124.97
Time + ARMA	5	139.49
LMB + Delay + AR	5	119.94*
LMB + Delay + MA	5	127.11
LMB + Delay + ARMA	6	126.16

Number of parameters and DIC for the models proposed for the number of DG2 cases. *: the selected model.

*Proposed models* - For each of the variables analysed, we proposed several variations of autoregressive integrated moving average (ARIMA) time series models (Chatfield 2016). However, given that the time series was not long enough to incorporate the full model, we used the simpler autoregressive moving average (ARMA) model that does not have the integrated noise term (I). The statistical models of the ARIMA family are the most common approach to study stochastic processes and to make forecasts using previous values from a time series (Chatfield 2016). These models do not make any assumption of the underlying mechanism of a given phenomenon being studied, but rather treats it as a stochastic process. Conversely, the ARMA model controls the autocorrelation and performs accurate measurements of the forecasting error (Chatfield 2016).

*Number of new cases of leprosy* - To test if the total number of leprosy cases exhibited a trend (i.e., the total number of cases increased or decreased with time), we proposed the following models:

*Null model* - the total number of cases was constant, and its observed variation was random following a negative binomial distribution.

$$C_{\left(t\right)}\;=\;K\;+\;\varepsilon$$ (1)

ε ~ N B (μ.α) (2)

Where *C(t)* is the number of cases at time *t* in years, *K* the annual average of cases, with e errors. *NB* is the negative binomial distribution with parameters *µ* (mean) and *α* (dispersion).

*Polynomial trend model* - The total number of cases followed a trend explained by a polynomy of an increased order beginning from one (linear trend).

$$C_{\left(t\right)}\;=\;\alpha_{0}\;+\;\alpha_{1}\;t\;+\;\alpha_{2}\;t^{{}_{2}}\;+\;...\;+\;\alpha_{n}\;t{\;}^{n}\;+\;\varepsilon_{t}$$ (3)

*ARMA (p, q) models* - The error of the model was autocorrelated, such that the observed number of cases was a function of the trend and a linear function of the past values. To model its dependence, we proposed an ARMA model (Chatfield 2016). The order of the autoregressive term (*p*) and the moving average (*q*) was restricted to one, given that the time series was short. This model was used in combination with the *Null* and *Polynomial* models.

$$C_{\left(t\right)}\;=\;c\;+\;\varepsilon_{t}\;+\;\sum_{i=1}^{p}\;\varphi_{i}C_{t-i}\;+\;\sum_{i=1}^{q}\theta_{i}\;\varepsilon_{t-i}$$ (4)

With *c* being the *trend* term which might be either the constant term of the null model (eq 1) or the polynom for the polinomial trend model (eq 3), φ_*i*_ are the parameters of the AR terms, and θ_*i*_ the parameters of the MA terms of the model.

*Proportion of MB cases of leprosy* - To test whether the proportion of MB leprosy cases exhibited a trend, we proposed the following models:

*Null model* - the proportion of cases was constant, and its observed variation was random. It contained only one parameter, the expected proportion, as in eq 1. However now, the errors followed a binomial distribution:

ε ~ B (n.p) (5)

With *n*, being the total number of *leprae* cases.

*Logit trend model* - the logit of the proportion followed a linear trend as a function of time. It contained two parameters, origin (*α*_*0*_) and slope (*α*_*1*_):

$$l_{\left(t\right)}\;=\;\alpha_{0}\;+\;\alpha_{1}t$$ (6)

Since the variable being tested was a proportion, a logit transformation was necessary to avoid expected values lower than zero or higher than one. Therefore, the expected number of MB cases is a logistic function of eq 6:

$$MB_{\left(t\right)}\;=\;\frac{\exp\;(l_{\left(t\right)})}{\exp\;(l_{\left(t\right)})\;+\;1}\;+\;\varepsilon_{t}$$ (7)

*ARMA (p, q) models* - As in the case of the previous variable, an ARMA model was proposed, in which the error of the model was autocorrelated, so the observed proportion of cases was a function of the trend and a linear function of the past values. The order of the autoregressive term (*p*) and the moving average (*q*) was restricted to one, given that the time series was short. This model was used in combination with the previous models.

*Number of new cases with grade 2 disabilities (DG2)* - To test whether the number of DG2 leprosy cases were a function of the number of MB cases and exhibited a trend (i.e., its proportion relative to the total number of cases increased or decreased with time), we proposed the following models:

*Null model* - The total number of cases was constant, and its observed variation was random following a negative binomial distribution, the model was similar to that of eq 1, with the same error distribution.

*Polynomial trend model* - The number of DG2 cases followed a trend explained by a polynomial of an increased order beginning from one (linear trend), as in eq 4.

*Delayed linear regression to the MB cases* - The number of DG2 cases was a linear function to the number of MB cases, with a time delay *d*_*t*_ expressed in years from the occurrence of MB cases to the registered DG2.

$$DG2_{\left(t\right)}\;=\;a_{0}\;+\;a_{1}C_{t-d}\;+\varepsilon_{t}$$ (8)

*ARMA (p, q) models* - The error of the model was autocorrelated, such that the observed number of cases was a function of the trend and a linear function of the past values. The order of the autoregressive term (*p*) and the moving average (*q*) was restricted to one, given that the time series was short. This model was used in combination with all the previous models.

*Parameter calculation and model selection procedure* - The parameters of the proposed models model were calculated using Markov Chain Monte Carlo (MCMC) method (Gelman et al. 2004), with the first 100,000 iterations discarded as a burn-in, and the following 100,000 iterations kept to calculate the *a posteriori* distribution of parameters and its information indexes, and to perform the ARIMA forecasting (see below).

Bayesian statistics require the proposal of a distribution of parameters expressing the initial belief or previous information of the values of each parameter, from which the random values of the MCMC procedure are drawn (Gelman et al. 2004), but we did not have strong *α priori* information. Therefore, we used uninformative *α priori* distributions that were always normal uninformative distributions with zero mean and a standard deviation of ten units.

The models proposed were of increasing complexity at steps of one extra parameter, beginning from the null model. If the model was accepted, another model with one extra parameter was proposed. The criterion of acceptance was to have a lower DIC.

*Software* - All MCMC calculations were performed using the *pymc* model for Bayesian statistics (Patil et al. 2010) for Python programming language.

*Projections of new cases of leprosy by 2020* - Since the proposed models contained ARMA models within them, they can be used to make forecasts (Chatfield 2016). The forecasts were made using the median of 100,000 projections using the parameters calculated in the Monte Carlo procedure on the selected model, and the credible interval of projections were calculated using the quantiles 2.5% and 97.5% of the ARMA credible intervals calculated for each Monte Carlo iteration.

*Ethics* - This study received approval from the Research Ethical Committee of the National Centre of Medical Genetics, Administración Nacional de Laboratorios e Institutos de Salud, ANLIS, Dr Carlos G Malbrán, Ministerio de Salud (Argentina), with the presence of the Coordinator Lic Maria Cecilia Luna. This committee certified that all data analysed were anonymous and accredited that the study presented no ethical concerns.

## RESULTS

**Fig. 2:**
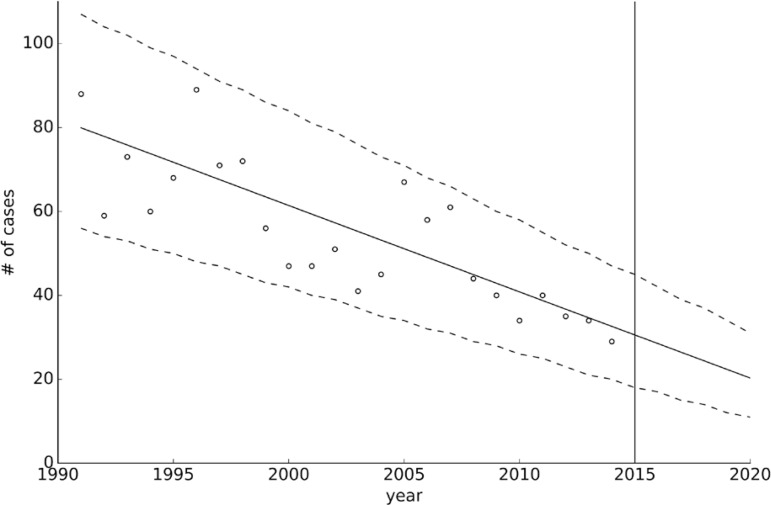
: number of cases of leprosy as a function of time. Circles indicate the observed number of cases, whereas the solid line is the median calculated trend (using the selected linear trend model) and the dashed lines its 95% credible intervals. Values to the right of the solid vertical line in 2015 are the forecasted values.

**Fig. 3:**
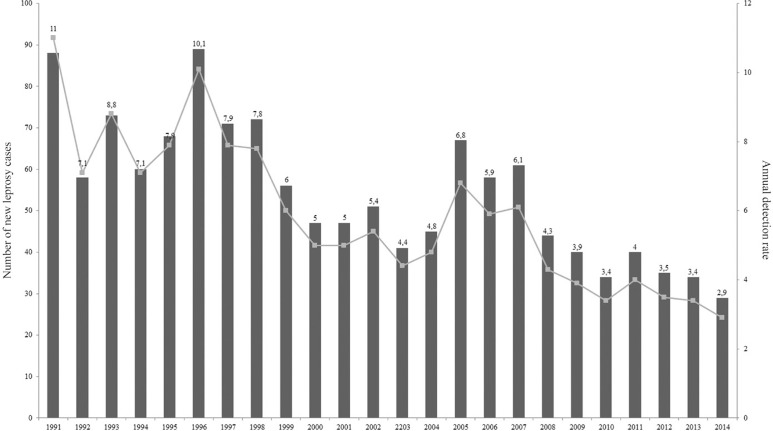
: time series of the number of new cases of leprosy and annual leprosy detection rates in Corrientes per 100,000 inhabitants, from 1991 to 2014. The indicators were based on population data determined by the population census of 1991, 2001, and 2010 and population estimates by Bureau of Statistics and Censuses of the Government of the Province of Corrientes (DEyC, Corrientes).

*Time series analysis: all leprosy cases* - The analyses of the 1308 new cases of leprosy from the data obtained from the Dermatological Clinic from 1991 to 2014, showed that the best model compromising explanatory power and complexity was the linear trend without autocorrelation (Table), with an origin ordinate near 78 (73.3-82.2) cases, and a slope of -2 cases/year. This means that the number of new cases of leprosy decreased over time and this trend was expected to continue in the near future (Fig. 2), evidenced by a sustained decrease in the detection rate from 11 cases/100,000 inhabitants in 1991 to 2.9 cases/100,000 inhabitants in 2014 (Fig. 3).

The analysis of the distribution of new cases by age group showed that the highest number of leprosy patients were aged 15-44 years (40.13%) and 45-64 years (38.83%), thus affecting the economically active population. Patients aged over 65 and 0-14 years represented 19.26% and 1.83% of new cases, respectively.

**Fig. 4:**
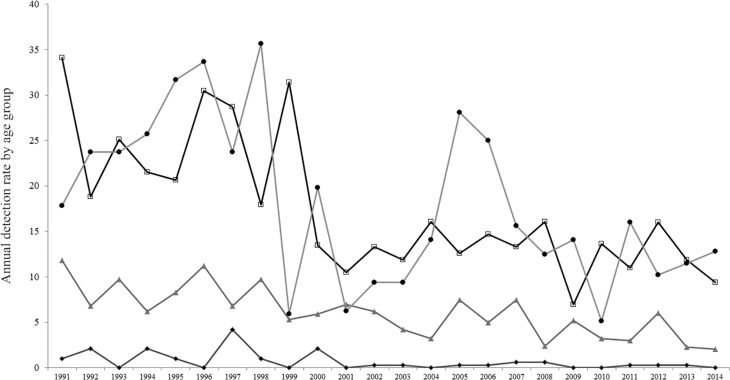
: annual detection rate by age group. Rhombus; indicates annual detection rate the of 0-14 year age group, triangles; 15-44; squares; 45-64 and circles; over (+) 65 years. The indicators were based on population data for each age group determined by the population census of 1991, 2001, and 2010 and population estimates by Bureau of Statistics and Censuses of the Government of the Province of Corrientes (DEyC, Corrientes).

**Fig. 5:**
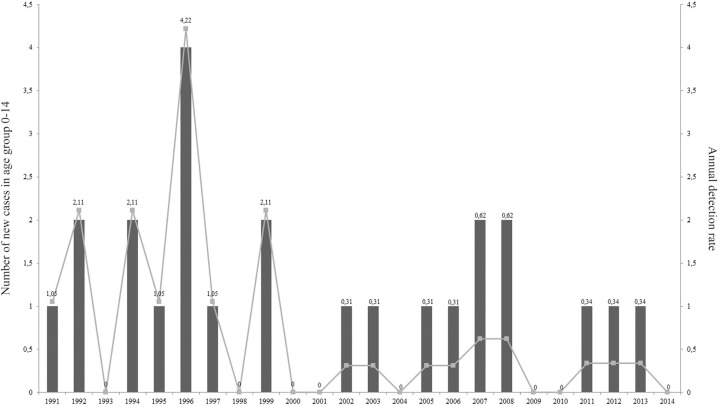
: annual detection rates in children 0-14 years old in Corrientes per 100,000 inhabitants from 1991 to 2014. The indicators were based on the population aged 0-14 years, determined by the population census of 1991, 2001, and 2010 of the Bureau of Statistics and Census of the province of Corrientes (DEyC), Argentina.

The highest average annual detection rate was evident in the age groups 45-64 years and over 65 years (17.83 and 18.2/100,000 inhabitants, respectively), followed by age groups 15-44 years (6.27/100,000 inhabitants) and 0-14 years (0.7/100,000 inhabitants) (Fig. 4). A time series analysis of age group 0-14, the most vulnerable population to leprosy, showed that the annual detection rate varied between 4.22/100.000 inhabitants in 1996 to 0 cases in the periods 2000-2001, 2009-2010 and 2014 (Fig. 5).

**Fig. 6:**
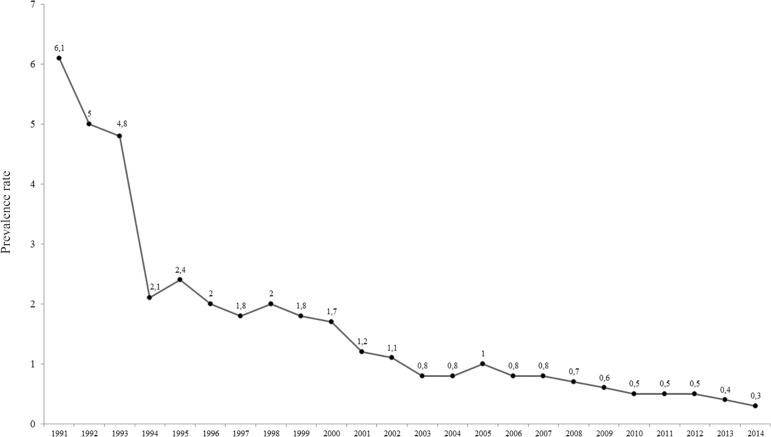
: time series of annual prevalence rate in Corrientes per 10,000 inhabitants from 1991 to 2014. The indicators were based on population data determined by the population census of 1991, 2001, and 2010 and population estimates by Bureau of Statistics and Censuses of the Government of the Province of Corrientes (DEyC, Corrientes).

The prevalence rate decreased sharply from 6.1/10,000 inhabitants in 1991 to 2.1/10,000 inhabitants in 1994, and then had a gradual and steady decline reaching 0.3/10,000 inhabitants in 2014, when considering all patients undergoing treatment that were living in Corrientes (Fig. 6).

**Fig. 7:**
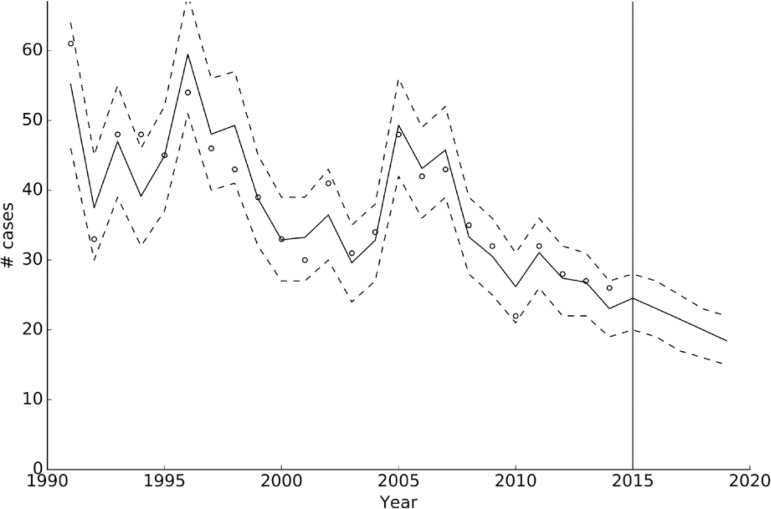
: number of multibacillary leprosy cases as a function of time. Circles indicate the observed number of cases, whereas the solid line is the median calculated trend (using the linear-logit trend model) and the dashed lines its 95% credible intervals. Values to the right of the solid vertical line in 2015 are the forecasted values.

*MB cases* - Concerning the operational classification established by the WHO with respect to the number of cutaneous lesions, we found that MB forms of leprosy predominated and represented 70.35% of all new cases studied. A negative trend was clearly evident when number of new MB cases of leprosy was plotted as a function of time, and the total number of cases may also be declining (Fig. 7).

**Fig. 8:**
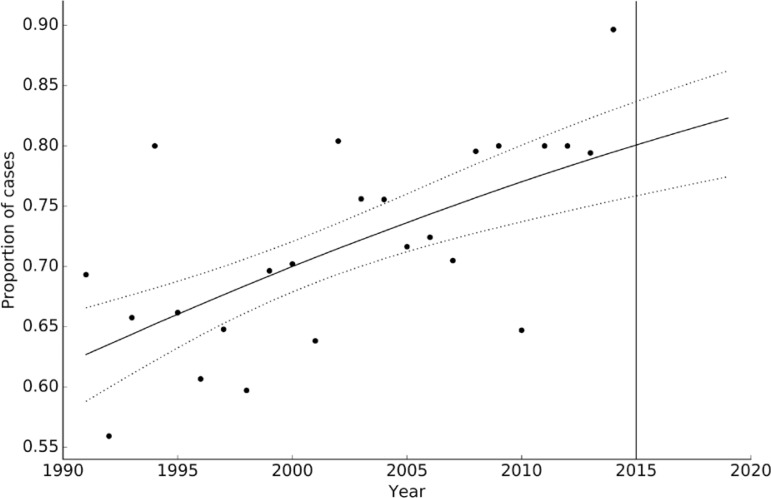
: proportion of multibacillary cases of leprosy as a function of time. Circles indicates the observed number of cases, whereas the solid line is the median calculated trend (using the same linear-logit trend model as in Fig. 7, but now shown as a proportion) and the dashed lines are the 95% credible intervals. Values to the right of the solid vertical line in 2015 are the forecasted values.

*Proportion of MB cases* - The model selection procedure for the proportion of MB leprosy cases resulted in the selection of a model in which the logit-transformation of the proportion of MB showed a linear trend (the trend followed a logistic function of time). Based on an origin ordinate of the logit transformation of 0.522 (0.318-0.732), slightly above 0.5, and a positive slope of 0.036 (0.018-0.054)/year, the results show that the proportion of MB cases increased continuously and will continue to increase in the short term (Fig. 8).

**Fig. 9:**
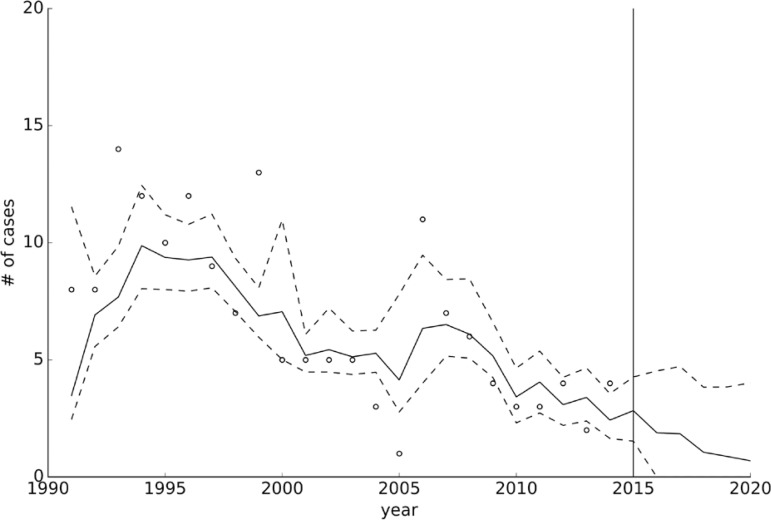
: number of DG2 cases as a function of time. Circles indicate the observed number of cases, whereas the solid line is the median calculated trend [using the selected AR(1) plus linear trend model, and delayed function to the number of multibacillary cases] and the dashed lines are the 95% credible intervals. Values to the right of the solid vertical line in 2015 are the forecasted values.

*DG2 trend* - The DG2 showed a more complex pattern when compared to the previous variables. The best model in terms of DIC was the one that followed a linear trend over time, had a linear relationship with the number of MB cases with a delay, and an order one autocorrelation, reflected in an ARMA (1,0) model for the residuals. The parameters of this model are shown in Table, showing a negative linear trend over time, and a slope as a function of MB cases that reveals that between 10 to 30% of the MB cases developed G2 disability/incapacity with a time delay between 0 (immediately) to 15 years. A slow decline was evident when this was plotted as a function of time, and it is expected to decrease more slowly in the following years, but with a wide estimation error in this forecast (Fig. 9).

## DISCUSSION

Leprosy is a chronic infectious disease caused by *M. leprae*, which can lead to physical disability, social stigma, and great suffering. Argentina is one of the Latin American countries with a high burden of leprosy, with more than one hundred new cases reported every year (WHO 2016). Despite this, in 2011 it was reported that Argentina had achieved the goal proposed by WHO towards the elimination of leprosy at the national level, but not at sub national one (OPS 2012).

Historically, Provincial programs are responsible for the control and actions reported pockets of moderate endemicity in the provinces of northeastern Argentina, including Corrientes, Entre Ríos and Misiones. However, there are no temporal studies of epidemiological indicators covering long timeframes that demonstrate present trends in the disease incidence.

This is a retrospective and quantitative study to analyse the dynamics of the epidemiological indicators of leprosy in the province of Corrientes between 1991 and 2014, and estimates the forecast prevalence by 2020. The data revealed a consistent decrease in the number of new leprosy cases from 1991 to 2014, evidenced by a sustained decrease in the annual detection rate from 11/100,000 inhabitants in 1991 to 2.9/100,000 inhabitants in 2014. Neighbouring provinces in which leprosy is also endemic reported an annual detection rate in the same period of 3.7/100,000 inhabitants in Misiones, 3.04/100,000 inhabitants in Chaco and 6.29/100,000 inhabitants in Formosa (SAL 2014). It is noteworthy that the province of Corrientes borders with the Republic of Paraguay, and constant migrations of the labour force between the two countries occurs. It is for this reason that patients are diagnosed and treated for leprosy, irrespective of their nationality and legal residential status, and are included in the statistics of Corrientes though not necessarily being natives of Argentina. This situation could influence the annual detection rate within this province (Ajalla et al. 2016). In any case, this finding is very encouraging. Considering that the study projections show that the number of new cases of leprosy are expected to continue on a downward trend in the future, we can infer that leprosy is truly decreasing.

In Corrientes between 1991 to 2014 patients aged 15-44 years, the main economically active population, and to a lesser extent those aged 45-64 years, were the most affected groups, representing 41.13% and 38.83% of cases, respectively. These findings are consistent with other reports and this suggests a negative effect on the economy, as this segment of the population may develop disabilities and reactional episodes that prevent them from working (Corrêa et al. 2012, PNCTyL 2012). Surprisingly, during the study period the highest average detection rate corresponded to patients in the age group over 65 years (18.2/100,000 inhab), followed by those aged 45-64 years (17.8/100,000 inhab). This novel finding implies that leprosy is belatedly diagnosed, either because patients do not attend the care health centre due to lack of information or economic resources, or because the symptoms are not recognised as leprous.

Leprosy in children aged 0-14 years is a strong indicator of recent transmission by active foci of infection. In Corrientes, the 0-14 year age group was the least affected, representing 1.83% of all new cases. The analysis showed that the annual detection rates in this vulnerable group were not uniform, reaching 0 in the periods 2000-2001, 2009-2010 and 2014 and increasing between 2008-2009 and 2011-2013. Given that the incubation period of leprosy varies from two to seven years, the increase in the number of children aged 0-14 years potentially exposes a lack of diagnostic cases by health services. Since detection rate replaced prevalence rate as a new indicator for monitoring leprosy elimination, this trend in the 0-14 year age group could indicate early contact with the bacillus, probably at home. Regarding the dynamic of this indicator, the Provincial Program for the Control of Leishmaniasis and Leprosy of Corrientes needs to intensify its surveillance and focus operational capacity in the active search for household contacts for the early diagnosis of the disease.

Corrientes is the first province in Argentina endemic for leprosy that was targeted to reach the goal of elimination of leprosy (WHO 1991) in 2010 (0.5 new cases/10,000 inhab), while Formosa (1.76/10,000 inhabitants) and Chaco (0.6/10,000 inhabitants) that were also endemic for leprosy did not reach this goal (PNCTyL, unpublished observations). However, the utility of the prevalence rate as an epidemiological indicator is still under discussion, as it is known that prevalence rate measures the disease burden on the health system rather than in the community, and its variation reflects mainly operational, rather than epidemiological trends (Penna & Penna 2012).

Cure of leprosy is dependent on proper diagnosis. MB clinical forms predominated in Corrientes, with a clear negative trend from 1991 to 2014 with projections indicating that the number of MB clinical forms will continue to decline by 2020. However, the proportion of MB cases increased in the same period and will continue to increase in the future. This is a characteristic of the stage of leprosy elimination and could be due to the use of multi-drug therapy (MDT) for several years, because drugs acts efficiently on susceptible strains of *M. le- prae* but genetically resistant strains persist (Lavania et al. 2014, Beltrán-Alzate et al. 2016). Considering that the proportion of MB cases represent cases at risk of complications, these findings imply the need to extend the chemotherapeutic coverage to all patients or to supply alternative drugs to those patients resistant to conventional treatment (Yamaguchi et al. 2016).

Interestingly, we found that between 10 and 30% of the MB cases developed DG2 in a period of 0 to 15 years. Moreover, while DG2 incidence declined slowly from 1991 to 2014, it is expected to decrease still more slowly in the future. However, the proportion of DG2 increased along the period and will continue to increase through to 2020. It is well known that one of the highest risk factors of disability is the ‘delay at detection’ and default in treatment (Alberts et al. 2011, Kumar et al. 2015); the proportion of DG2 is an indicator of the quality of case detection activities.

Corrientes is the first province endemic for leprosy in Argentina, which has not only improved its epidemiological indicators but is predicted to continue improving them in the future. However, projections indicate a constant increase in the proportions of MB cases and DG2. In relation to these indicators, the Provincial Program for the Control of Leishmaniasis and Leprosy of Corrientes needs to work to eradicate leprosy definitively.
